# A study protocol for an observational cohort investigating cardiac transthyretin amyloidosis flow reserve before and after Tafamidis treatment: The AMYTRE study

**DOI:** 10.3389/fmed.2022.978293

**Published:** 2022-08-23

**Authors:** Bastien Vançon, Arnaud Bisson, Maxime Courtehoux, Anne Bernard, Matthieu Bailly

**Affiliations:** ^1^Nuclear Medicine Department, CHR Orleans, Orléans, France; ^2^Cardiology Department, CHR Orleans, Orléans, France; ^3^Cardiology Department, CHRU Tours, Tours, France; ^4^EA4245 T2i, Tours University, Tours, France; ^5^Nuclear Medicine Department, CHRU Tours, Tours, France; ^6^UMR 1253, iBrain, Université de Tours, Inserm, Tours, France

**Keywords:** myocardial blood flow, myocardial flow reserve, CZT SPECT, amyloidosis, Tafamidis

## Abstract

**Introduction:**

Anginal symptoms and signs of ischemia have been reported in some patients with cardiac transthyretin amyloidosis (ATTR) without obstructive epicardial coronary artery disease (CAD). Few studies found that coronary microvascular dysfunction was highly prevalent in subjects with cardiac amyloidosis, even in the absence of epicardial CAD. The purpose of this study is to confirm the coronary microvascular dysfunction, and to go further with evaluation of the effect of Tafamidis on microvascular dysfunction after 24 months of treatment.

**Methods and analysis:**

This study is a multicentric, prospective, observational cohort study. Adult patients with confirmed ATTR cardiomyopathy seen in the nuclear medicine departments of three large referral centers and treated with Tafamidis will be included. At baseline, patients will have a clinical and echocardiography evaluation. They will undergo a dynamic rest/stress cardiac scintigraphy with flow and reserve measurements before and 24 months after Tafamidis introduction. The primary outcome of this study will be the variation of stress and rest myocardial blood flow and flow reserve between baseline and 24 months after treatment. The effect of Tafamidis will be assessed by an intention to treat analysis.

**Ethics and dissemination:**

The study has received the following approvals: Orleans Hospital Research Committee (CHRO-2021-05) and Sud-Mediterranée IV Regional Ethics Committee (21 06 02). Results will be made available to physicians, the funders, and other researchers.

**Clinical trial registration:**

[https://clinicaltrials.gov/ct2/show/NCT05103943], identifier [NCT05103943].

## Introduction

### Context

Systemic amyloidosis represents rare conditions in which the gradual extracellular accumulation of insoluble fibrillar proteins disrupts the structure of the affected tissues and causes organ impairment. Transthyretin amyloidosis (ATTR) commonly affects the heart. The amyloid deposits cause an increase in ventricular wall thickness and result in restrictive cardiomyopathy presenting primarily as biventricular congestive heart failure. Ischemia signs and anginal symptoms have been described in some individuals with cardiac amyloidosis and without obstructive epicardial coronary disease (CAD) (1–3).

### Current knowledge

Dorbala et al. found that coronary microvascular dysfunction was highly prevalent in patients with cardiac amyloidosis, even in the absence of epicardial CAD, and might explain their anginal symptoms. They found lower stress and rest myocardial blood flow (MBF) and lower myocardial flow reserve (MFR) in their cardiac PET study (using ^13^N), including 21 patients with definite cardiac amyloidosis without epicardial CAD (4). Moreover, reduction of MFR during cardiac amyloidosis has also been described using myocardial contrast echocardiography (5).

The use of cadmium zinc telluride (CZT) detectors in SPECT technology allows acquisition in a non-rotating mode and therefore acquisition of time–activity curves, and improves sensitivity and temporal resolution (6). Thus, absolute MBF and MFR estimation by SPECT becomes possible as shown in experimental animal models (7) and also in clinical studies with comparison to PET (8–11), with a good correlation between those two techniques. SPECT myocardial perfusion imaging (MPI) showed, in a recent sub-study of the PACIFIC trial, a low per-vessel sensitivity [42%, confidence interval (CI): 34–50%] in comparison to PET (81% CI: 72–87%) and a low per-patient sensitivity for detection of CAD: 61% (CI: 48–72%) vs. 90% (CI: 80–96%), respectively, for SPECT and PET. However, specificity was 93% (CI: 85–97%) and 87% (CI: 78–93%) for SPECT and PET. Using the entire cohort of patients and vessels, and regarding the diagnostic performances of imaging modalities for significant CAD with an intention-to-diagnose, per-patient area under the receiver operating characteristic curve and diagnostic accuracy were outperformed by PET (0.90 and 86%; *p* = 0.005 and *p* < 0.001, respectively) in comparison to SPECT (0.74 and 76%, *p* = 0.087 and *p* = 0.266, respectively) (12). Moreover, PET imaging enables higher spatial resolution and enhanced image quality (13). Thus, PET has become a reference, also thanks to added parameters in PET in comparison with SPECT: dynamic PET and systematic CT that enables calcium scoring. MBF and MFR incrementally improve diagnostic and prognostic accuracy over MPI alone. 82-Rubidium or 13N-Ammonia PET imaging with pharmacological stress MPI, MBF, and MFR can be acquired simultaneously without incremental cost, radiation exposure, or significant processing time (14). Nuclear cardiology clinics with PET access have incorporated MBF quantification into clinical routine, but this is not the case for conventional SPECT MPI. However, especially in Europe, cardiac PET remains of difficult access in comparison to SPECT and now CZT SPECT.

New therapeutic options are arising in cardiac ATTR. In a multicentric, international, double-blind, placebo-controlled, phase 3 trial, including 441 patients with ATTR cardiomyopathy, Maurer et al. showed that Tafamidis (80 mg and 20 mg) was associated with reductions in all-cause mortality (78 of 264 [29.5%] vs. 76 of 177 [42.9%]; hazard ratio, 0.70; 95% confidence interval [CI], 0.51–0.96) and cardiovascular-related hospitalizations after 18 months [relative risk ratio of 0.68 (0.48 per year vs. 0.70 per year; 95% CI, 0.56–0.81)], and reduced the decline in functional capacity and quality of life after 6 months as compared with placebo in patients with transthyretin amyloid cardiomyopathy (15). Tafamidis has now been approved for patients with ATTR cardiomyopathy and is recommended class I in the latest European guidelines on heart failure (16).

In APPOLO trial (international, randomized, double-blind, placebo-controlled phase 3 trial with hereditary ATTR amyloidosis), Patisaran, by stopping the synthesis of TTR through gene silencing, decreased mean left ventricular wall thickness, global longitudinal strain, N-terminal prohormone of brain natriuretic peptide, and adverse cardiac outcomes compared with placebo at month 18 (17). Authors used Patisaran 0.3 mg/kg IV once every 3 weeks in 225 ATTR-FAP patients. Others pharmacological trials explored Inotersen treatment (gene silencer too), but they focused on neurological disease: NEURO-TTR phase 3 trial (18).

About others tetramer stabilizers, no beneficial cardiac effect has been shown in Diflunisal treatment, and a randomized double-blind, placebo-controlled Phase 2 trial has been initiated, as mentioned in a recent mini review (19).

To our knowledge, no study focused on the effect of cardiac amyloidosis treatment on microvascular dysfunction.

### Study and aims

We hypothesize that Tafamidis could improve microvascular dysfunction in patients suffering from ATTR cardiomyopathy, quantifiable with MBF and MFR. This could result into a MBF and MFR improvement at the evaluation after 24 months of treatment, in comparison with baseline. The second minor objective is to confirm the microvascular dysfunction described in PET by Dorbala et al., using dynamic SPECT.

## Methods and analysis

### Study protocol

This is a multicentric, prospective, observational cohort study involving three French academic hospitals (University Hospital of Angers, University Hospital of Tours and Regional Hospital of Orleans). Study patients will enter the cohort after they have been diagnosed with cardiac ATTR, including ^99m^Tc-PYP scintigraphy (grade 2 or 3) (20–22), according to the diagnostic algorithm for cardiac ATTR proposed by Gillmore et al., after they meet inclusion/exclusion criteria (Table 1).

**TABLE 1:** Inclusion and exclusion criteria.

Inclusion criteria	Exclusion criteria
● Patients aged from 18 to 90 years	● Other heart failure cause (not ATTR)
● Understanding and speaking French ● With TTR amyloid cardiomyopathy (ATTRxt or ATTRm) confirmed by the association of: ⚬ clinical heart failure (NYHA I-III) ⚬ with echocardiogram and/or magnetic resonance imaging suggesting/indicating cardiac amyloidosis, ⚬ grade 2 or 3 99mTc PYP or bone scintigraphy, ⚬ and negative biological findings (i.e., serum immunofixation, urine immunofixation, serum free light chain assay), or, if one of those criteria is not met, amyloid deposits on biopsy specimens (from cardiac and non-cardiac sites), ● Intention to treat (Tafamadis)	● New York Heart Association (NYHA) class IV heart failure. ● Light-chain amyloidosis ● Liver or heart transplantation history ● Glomerular filtration rate below 25 mL per minute per 1.73 m^2^ of bodysurface area (Cockroft) ● High liver transaminase levels (two times the upper limit of the normal range) ● Severe malnutrition defined by a modified body-mass index (mBMI: serum albumin level in grams per litter multiplied by the conventional BMI) of less than 600; ● Concurrent treatment with non-steroidal anti-inflammatory drugs, tauroursodeoxycholate, doxycycline, calcium-channel blockers, digitalis, or ticagrelor ● Previous treatment with tafamidis or patisaran ● Previous CAD, severe epicardial stenosis with revascularization or ticagrelor treatment, coronary artery bypass grafting, myocardial infarction ● Contra-indications to pharmacological stress testing MPI: severe hypotension (<90 mmHg of Systolic arterial pressure), atrioventricular block 2nd or 3rd grade, carotid stenosis (unilateral > 70%, bilateral > 50%) ● Pregnancy ● Breastfeeding ● Protected adults

### Inclusion and exclusion criteria

Criteria are summarized in Table 1.

### Consent

Patients who meet all inclusion criteria and none of the exclusion criteria will be offered the opportunity to participate in the study and provided with all the relevant information verbally and in writing.

The free and informed consent, in writing, of the potential participant will be obtained by the investigator before the inclusion of this person in the research.

### Interventions and participant timeline

**FIGURE 1:**
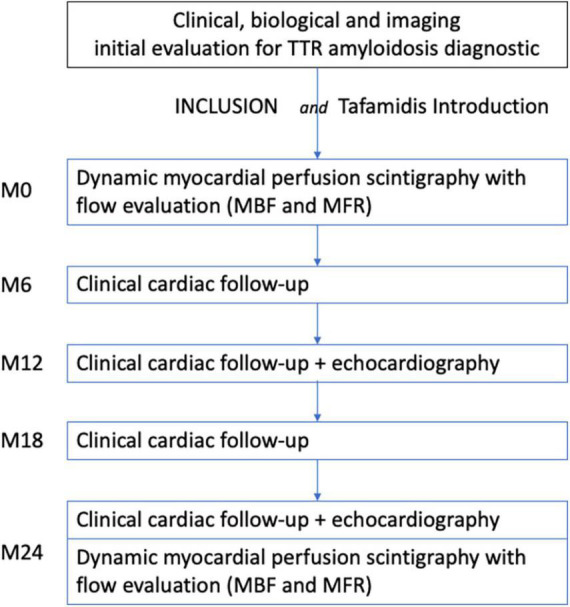
Flow of the participants throughout the study. MBF, Myocardial Blood Flow; MFR, Myocardial Flow Reserve.

The schedule of participants’ enrolment, interventions and assessments are presented in Figure 1.

Potential participants referred for ATTR bone or PYP scan will be pre-screened by the nuclear medicine physician. After confirmation of TTR cardiac amyloidosis diagnosis, including ^99m^Tc-HDP or ^99m^Tc-PYP scintigraphy (grade 2 or 3) (20, 22, 23) according to the diagnostic algorithm for cardiac ATTR amyloidosis proposed by Gillmore et al., patients will be included and Tafamidis introduced, according to the guidelines. The treatment will be fully reimbursed by health insurance in this indication.

We estimate that the recruitment will take around 12–24 months. Participants will not receive any financial or non-financial incentives.

**FIGURE 2:**
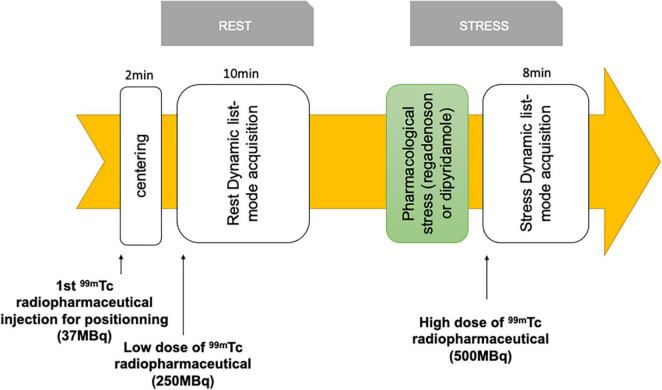
Dynamic scintigraphy protocol.

Patients will be included before the baseline dynamic CZT-SPECT myocardial perfusion imaging (with MBF and MFR), after written consent. Dynamic SPECT imaging protocol is displayed in Figure 2. All acquisitions will be performed on the same system: Discovery NM530c cardiac CZT cameras (General Electric Healthcare, Haifa, Israel) in all departments. The radiopharmaceutical used will be either ^99m^Tc-tetrofosmin for Orleans and Tours, and ^99m^Tc-mibi for Angers. Initial injection of 37 MBq will be used to center the patient’s heart in the field of view. Rest dynamic acquisition will then be performed with an injection of 250MBq of ^99m^Tc radiopharmaceutical, then flushed by 50 mL of saline to ensure consistent delivery of a tight bolus. Stress will then be induced using either a regadenoson (400 μg) injection or a dipyridamole perfusion (0.56 mg/kg), immediately followed with a 500MBq of ^99m^Tc radiopharmaceutical injection at hyperemia peak. Dynamic SPECT data will be reconstructed using Corridor 4DM™ software (INVIA, Ann Arbor, MI, United States) on a Xeleris workstation (General Electric Healthcare, Haifa, Israel).

Epicardial obstructive CAD will be ruled out in subjects with pathological initial imaging (coronary angiography or cardiac CT depending on the cardiologist choice). Patients with epicardial stenosis will be analyzed apart, especially if revascularization or ticagrelor treatment is performed.

Visits are mainly already included in the cardiologic follow-up of the Tafamidis treatment at 6, 12, 18, and 24 months (with cardiac echography at 12 and 24 months). Clinical data and echocardiography findings will be resumed. Clinical data will include blood pressure, NYHA class, Kansas City Cardiomyopathy Questionnaire-Overall Summary (KCCQ-OS), 6-min walking test, electrocardiogram, any treatment interruption, or hospitalization. Cardiac echography will include at least: left ventricles volumes measurement, ejection fraction, wall thickness, strain results.

Last evaluation will be CZT SPECT MPI after 24 months of treatment, in the same conditions as baseline.

### Trial outcomes

The primary outcome is the variation of stress and rest MBF and MFR assessed by dynamic SPECT, between baseline and 24 months after Tafamidis treatment.

The secondary outcomes are:

• Results from baseline dynamic SPECT, confirming the stress and rest MBF and MFR reduction in cardiac ATTR patients,
• Results from echo/MRI.

### Statistical analysis plan

Interim analysis is planned after all inclusions for MBF and MFR baseline evaluation. Final analysis is planned after completion of study. A subgroup with patients suffering from CAD, assessed by invasive coronary angiography or cardiac-CT after baseline SPECT will be analyzed apart.

All MBF and MFR values will be described as mean ± SD. Normal distribution of the value will be evaluated. Comparisons between continuous variables will be performed using two-tailed paired *t*-test. Non-parametric variables will be listed as medians and compared using the Mann–Whitney *U* test. Discrete variables will be described as proportions and compared with the chi-square test. Correlations will be performed using Pearson R or non-parametric methods (Spearman rho) as indicated. Multivariable linear regression analyses will be performed to study the independent contributions of various parameters on stress MBF and MFR.

The total number of patients expected in this “proof of concept” study is 50 patients. In the context of a pilot study, the workforce calculation is not applicable, as frequently in the case of exploratory studies on innovative technologies (24). This number of patients is also compatible with the recruitment capacities of the three investigative centers.

### Data management

Data will be collected electronically including eligibility, baseline clinical, biological and imaging characteristics, treatment and outcome details. Every site will have access to the electronic case report forms *via* a web-based data collection system created using EOL (developed by Medsharing). Investigators will be given instructions for using this tool.

All trial documents will be archived and securely kept for 15 years after study completion.

### Trial organization structure

The Centre Hospitalier d’Orléans will be the sole sponsor of this multi-center study and will ensure the safety and respect of individuals who have agreed to participate in the trial. The sponsor has a quality assurance system in place to monitor the implementation of the study at the research centers.

Clinical Research Associates (CRA) will be appointed by the sponsor. After an initial visit, their main role will be to make regular follow-up visits at the different study sites.

The purpose of study monitoring, as defined in the Good Clinical Practices (GCP section 5.18.1), is to verify that:

• Research subjects are safe, protected, and their rights are respected.
• The data recorded are accurate, complete, and consistent with the source documents.
• The study is conducted in accordance with the current version of the protocol, GCP and all legal and regulatory requirements.

For this study, the appropriate monitoring level was determined based on the complexity, impact, and budget for the study. Therefore, the sponsor, in agreement with the coordinating investigator, agreed on a logistical score and impact and the corresponding study monitoring level of: A level.

A CRA assigned by the sponsor will oversee the proper conduct of the study, the collection, documentation, recording, and reporting all handwritten data, in accordance with the Standard Operating Procedures applied within the research center and in accordance with Good Clinical Practice and legal and regulatory requirements.

The investigator and his/her team agree to be available for regular Quality Control visits by the CRA. During these visits, the following points will be examined: written consent, compliance with the study protocol and its procedures, quality of the data collected in the case report forms: accuracy, missing data, consistency of the data with the “source” documents (medical files, appointment books, original copies of laboratory results, etc.), management of the treatments used.

### Confidentiality statement

Confidential trial data will be stored in accordance with the General Data Protection Regulation 2018. Patients will only be identified using only their date of birth and unique trial ID number.

The processing of personal data for this research falls under the scope of the provisions of Articles 53–61 of the Law of January 6, 1978 relating to information technology, data files and privacy, modified by Law No. 0204-801 of August 6, 2004.

All personal data for this trial will be processed in accordance with Chapter IX of the amended French Data Protection Act of January 6, 1978 (articles 53–61).

### Trial status

Recruitment for the trial has been opened in February 2022.

## Discussion

AMYTRE study aims to evaluate a potential effect of Tafamidis treatment on microvascular dysfunction in patients with cardiac ATTR, assessed by dynamic SPECT.

As we previously described, microvascular dysfunction has been found using PET (4), but never confirmed with dynamic SPECT. Dynamic CZT myocardial SPECT has now become a more available tool, showing similar results to PET in terms of MFR evaluation (8–11).

A potential bias in this study will be the finding of authentical CAD after initial SPECT. In this case, as described, patients will undergo coronary angiography or cardiac CT. If an epicardial stenosis is found, and requires treatment, patient will be analyzed in a subgroup. Indeed, MFR might be affected by revascularization or ticagrelor. Aikawa et al. reported in a prospective multicentric observational PET ^15^O-water study a significant improvement of PET MFR 6 months after percutaneous coronary intervention (PCI) or coronary artery bypass grafting (CABG) in patients with abnormal baseline MFR (<2.0), but no significant effect of optimal medical therapy alone (25). Driessen et al. also confirmed a significant improvement of stress MBF assessed by ^15^O-water PET in patients with PCI, even superior compared with CABG (12). Other studies showed an increase of global and regional MBF and MFR in CAD patients treated with ticagrelor when compared to clopidogrel (26, 27).

The other potential bias are diabetic patients. It has been extensively reported that diabetic patients can have microvascular dysfunction, resulting in reduced stress MBF and MFR (28, 29). Clinical informations about diabetic patients (time of evolution, known complications such as diabetic nephropathy, retinopathy…) will be collected, to better rule out any potential confounding factor.

With this study, we aim to demonstrate an impact of Tafamidis on microvascular dysfunction. Regarding our secondary endpoint, various perspectives can be seen. Firstly, the absence or low proportion of microcirculatory dysfunction in patients with cardiac amyloidosis. However, the sample size remains small, and the evaluation may be too early compared with the diagnosis (although often late); this is therefore a possible limitation of our study, which may explain the failure to find this microcirculatory dysfunction. The second option is a high proportion of microcirculatory dysfunction in patients with cardiac amyloidosis. Our hypothesis would then be confirmed and will therefore be in line with the study of Dorbala et al. performed in PET (4). Other studies also found microcirculatory dysfunction in these patients, as well as in other infiltrative heart diseases such as sarcoidosis (30), which reinforces the probability of this option.

Regarding the variation in MFR values after treatment, the first possibility is a significant decrease of MFR. In this case, the primary hypothesis of an improvement in microcirculatory dysfunction after treatment with Tafamidis would then not be verified. Thus, the lack of effect of Tafamidis on microcirculatory damage would be considered, and the benefits on overall survival, quality of life and cardiovascular events mentioned in the previous studies would probably be due to another pathophysiological process. However, an aggravating factor of Tafamidis on microcirculatory function could not be raised in our study in the absence of a “control” population (untreated or benefiting from another treatment), so another study with this objective could be envisaged, in order to distinguish the natural evolution of the pathology from a treatment effect.

We might also consider a significant improvement of MFR after treatment. This would confirm a potential pathophysiological mechanism of the clinical improvement obtained by Tafamidis. It would then be interesting, in a second study, to evaluate whether this improvement in coronary microcirculation occurs early, within the first 12 months. Indeed, there is a need for biomarkers to predict the response to Tafamidis. Some will not respond to therapy; Monteiro et al. built a model to predict responsiveness to Tafamidis (31). They identified that disease severity, sex, and native TTR concentration at the outset of treatment were the most relevant predictors of response to Tafamidis. Plasma Tafamidis concentration after 12 months of therapy was also a predictor of response for male patients. Falk et al. suggested that measurement of TTR level change post-Tafamidis might be a surrogate for stabilization and could be an accurate measure of drug efficacy (32). Other treatments such as RNA interference therapeutics are developing rapidly in this indication with, it seems for the moment, great efficacy (17). So, the idea of a biomarker, possibly early, of efficacy would make it possible to quickly orient patients labeled as non-responders toward a switch to another therapy or to an association (combo RNAi and Tafamidis).

Finally, we might also envisage a lack of significant change in MFR values between 0 and 24 months. Our primary hypothesis would be untested. The potential limitations of our study would be a too short follow-up, or a too small sample.

## Ethics and dissemination

The trial will be performed in accordance with the recommendations guiding physicians in biomedical research involving human subjects, adopted by the 18th World Medical Association General Assembly, Helsinki, Finland and stated in the respective participating countries laws governing human research, and Good Clinical Practice. The protocol was approved by Orleans Hospital Research Committee (CHRO-2021-05) and by Sud-Mediterranée IV Regional Ethics Committee (21 06 02); current version in use is 3.0.

After the end of the trial, the collaborators will discuss the main results prior to publication. The scientific communications and reports corresponding to this study will be produced under the responsibility of the principal investigator coordinating the study with the agreement of the responsible investigators and will be submitted for publication in peer-reviewed journals. Manuscripts will be prepared, and authorship will be determined by mutual agreement.

## Ethics statement

The studies involving human participants were reviewed and approved by Sud-Mediterranée IV Regional Ethics Committee. The patients/participants provide their written informed consent to participate in this study.

## Author contributions

BV drafted the manuscript. MB, ABi, and ABe revised the manuscript critically for important intellectual content. All authors have contributed to the work and have read and approved the manuscript.
